# Supplementary material for: Control of protein palmitoylation by regulating substrate recruitment to a zDHHC-protein acyltransferase
Source: Commun Biol. 2020 Jul 31;3:411. doi: 10.1038/s42003-020-01145-3 (PMC7395175; doi:10.1038/s42003-020-01145-3)

Figure 1a

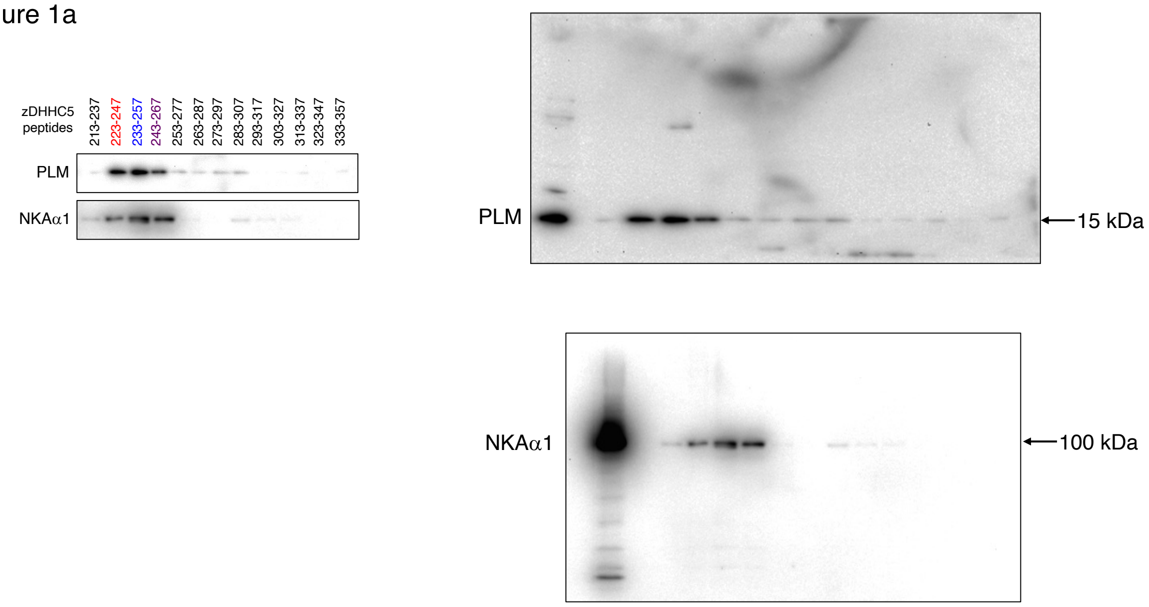

Figure 2a

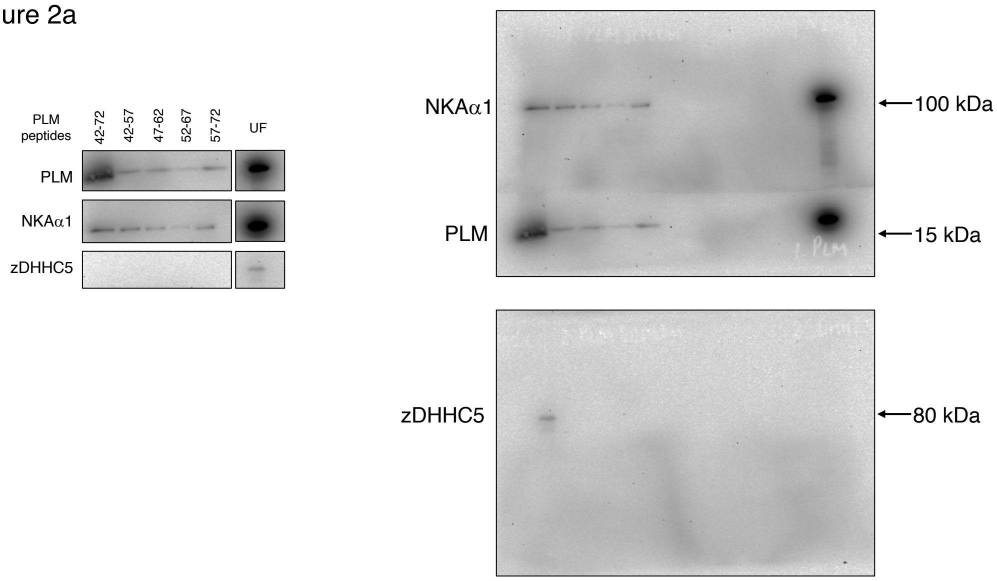

Figure 2b

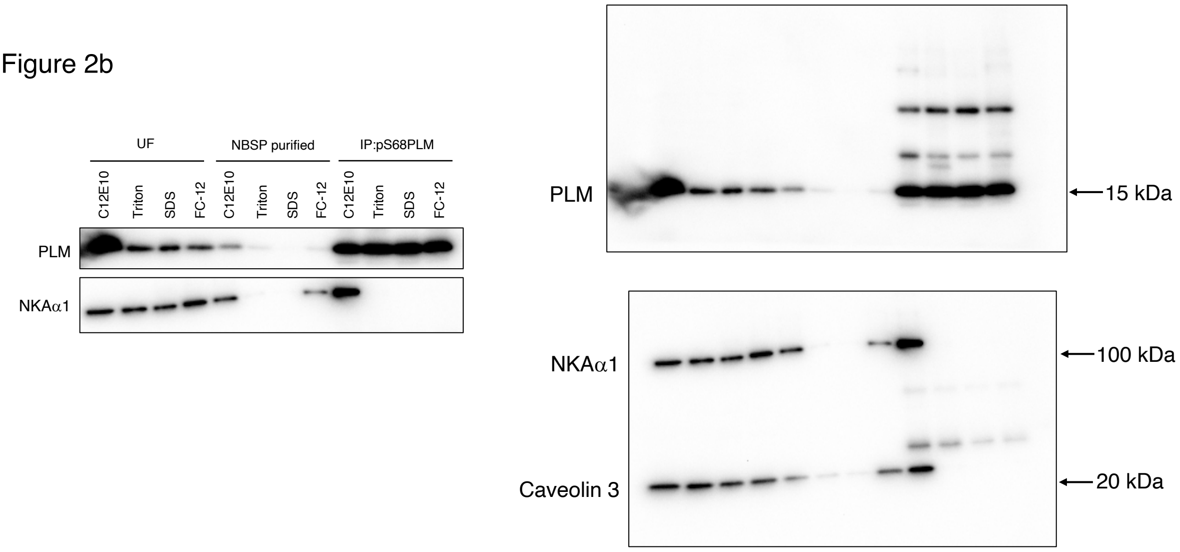

Figure 2c

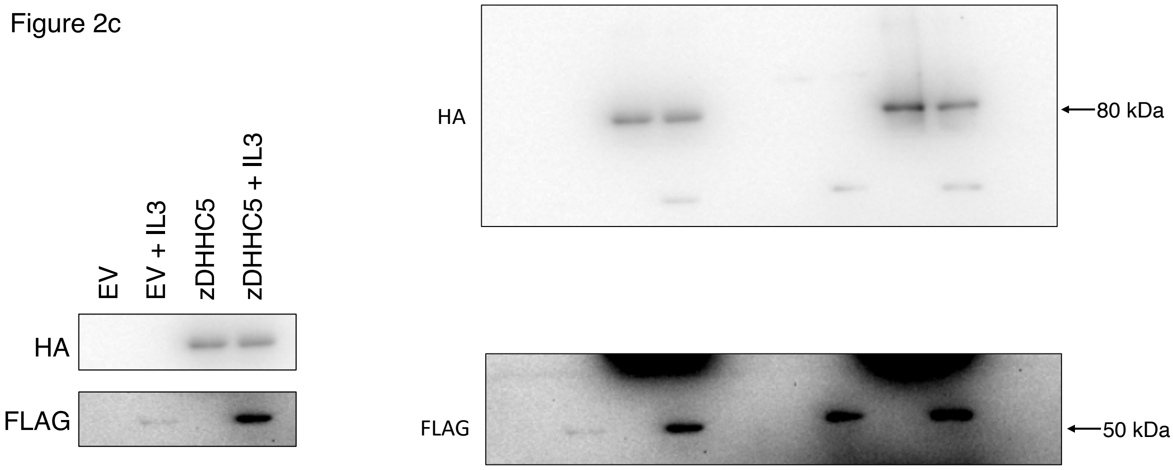

Figure 2d

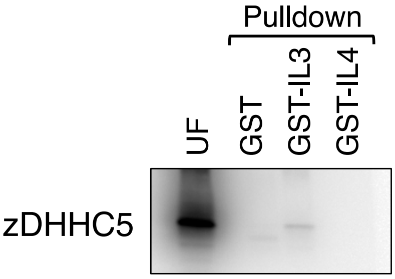

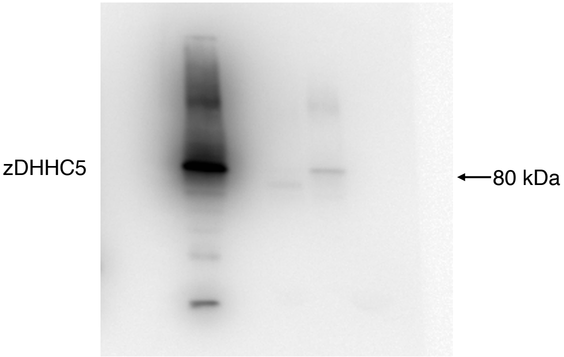

Figure 3a

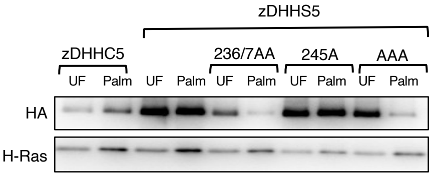

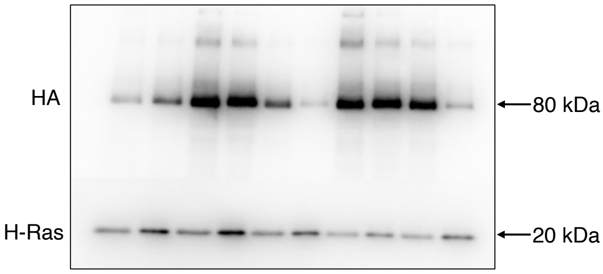

Figure 3b

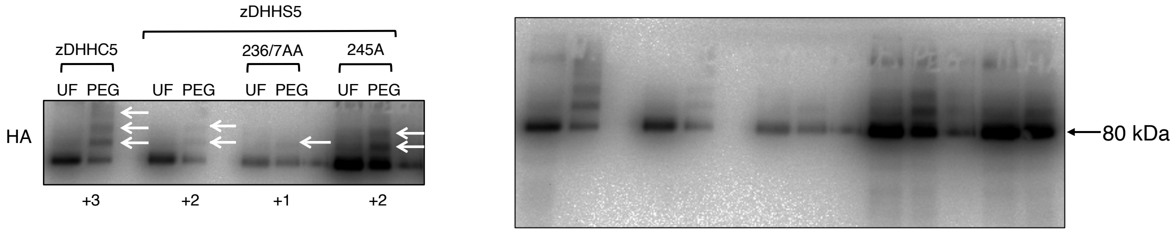

Figure 3c

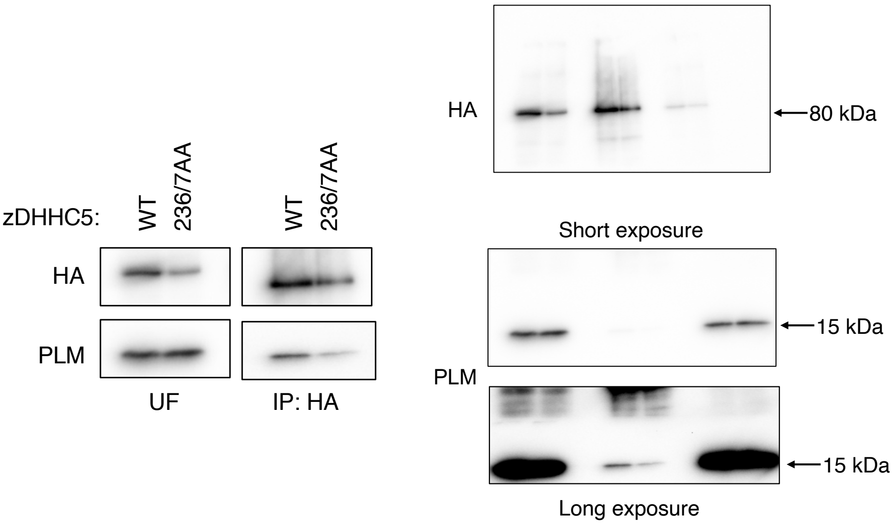

Figure 3d

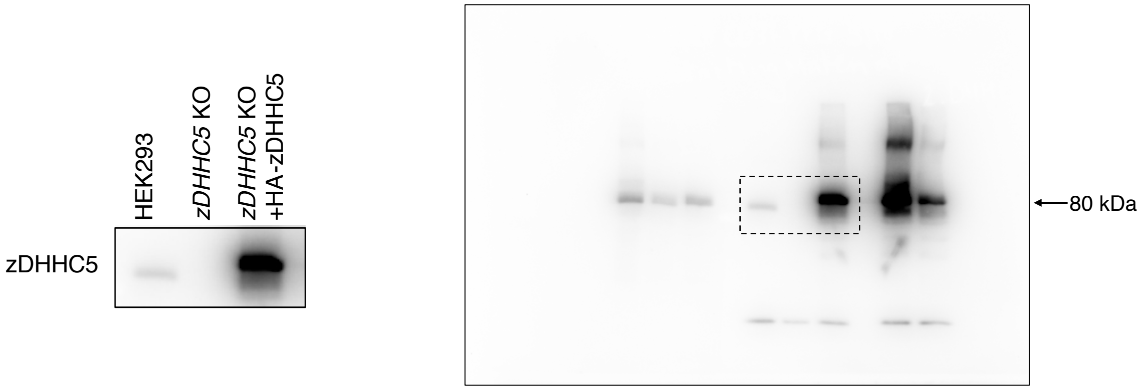

Figure 3e

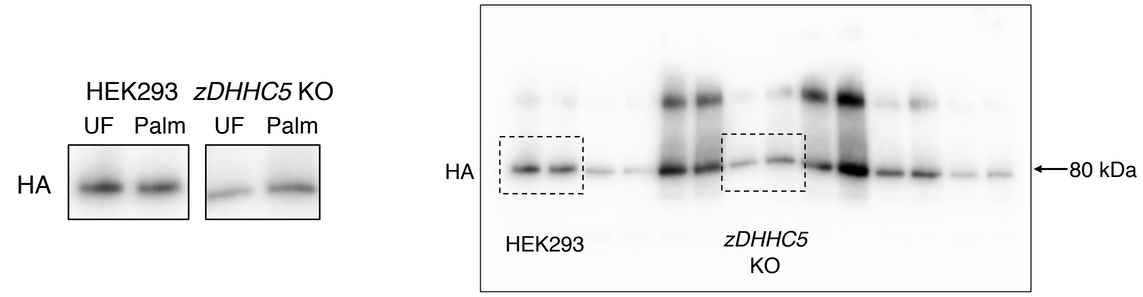

Figure 3f

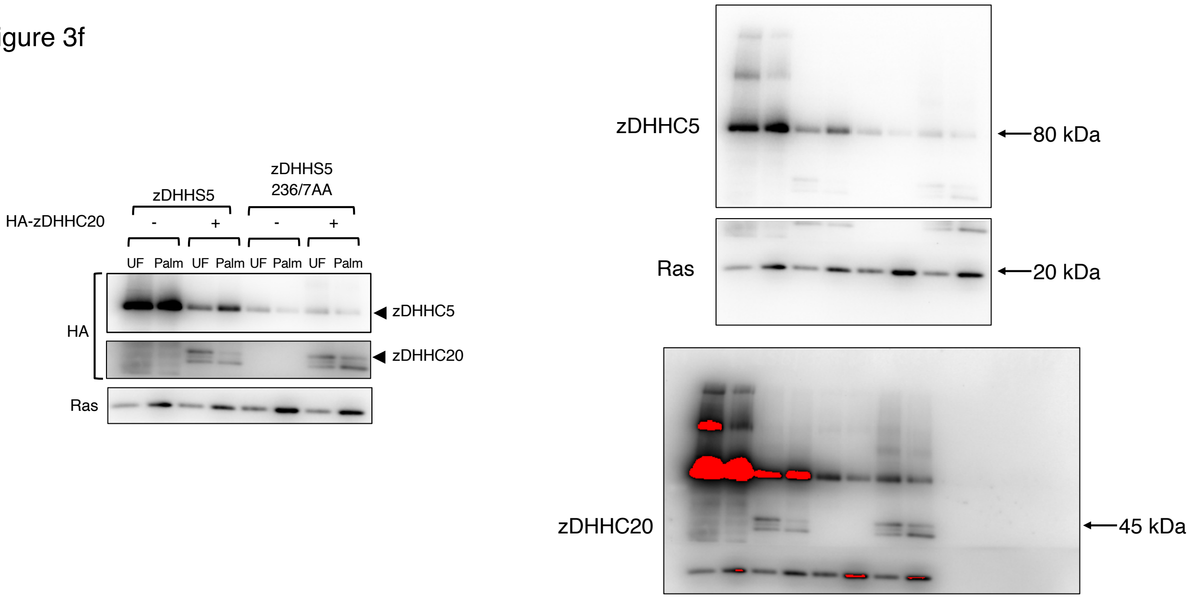

Figure 3g

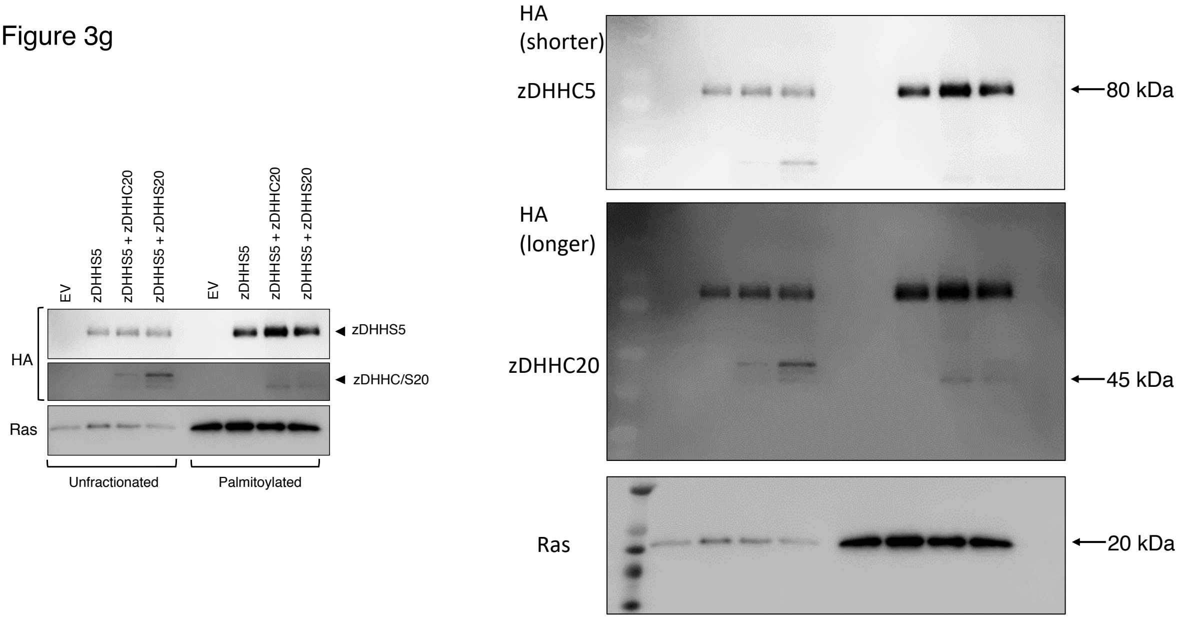

Figure 3h

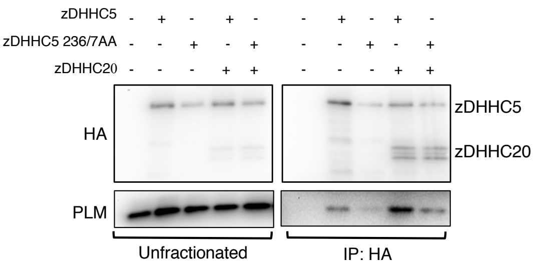

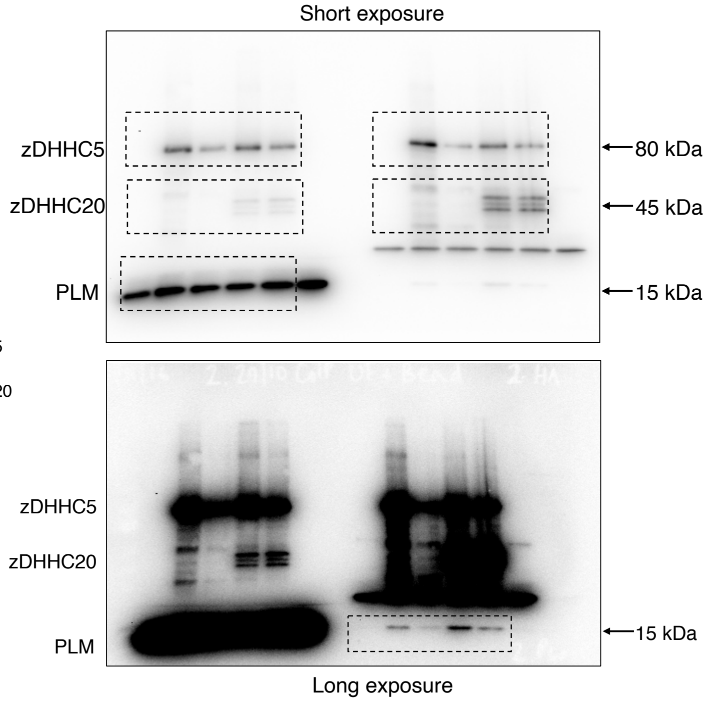

Figure 4a

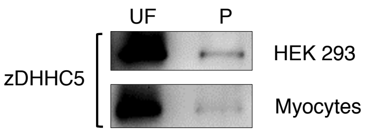

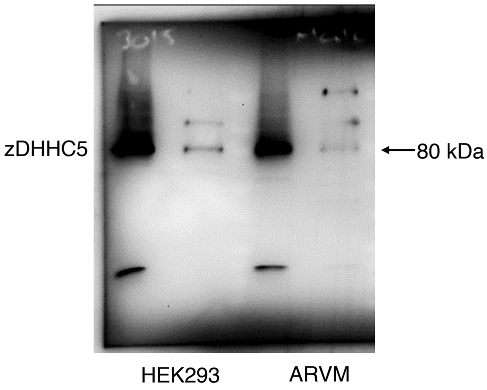

Figure 4b

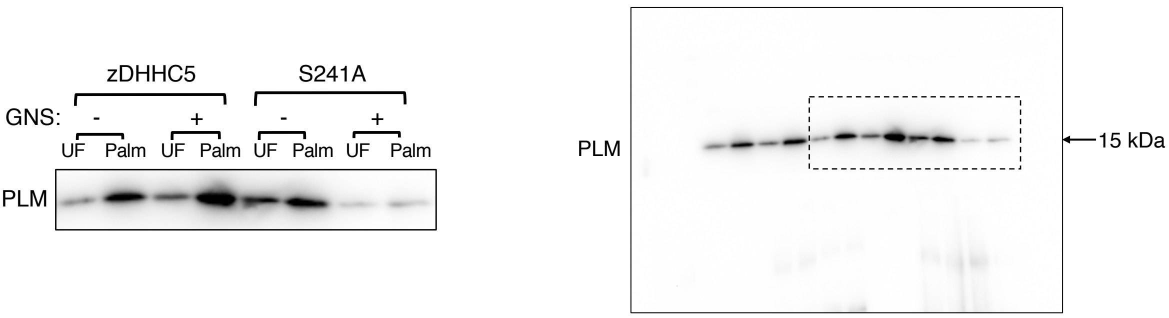

Figure 4c

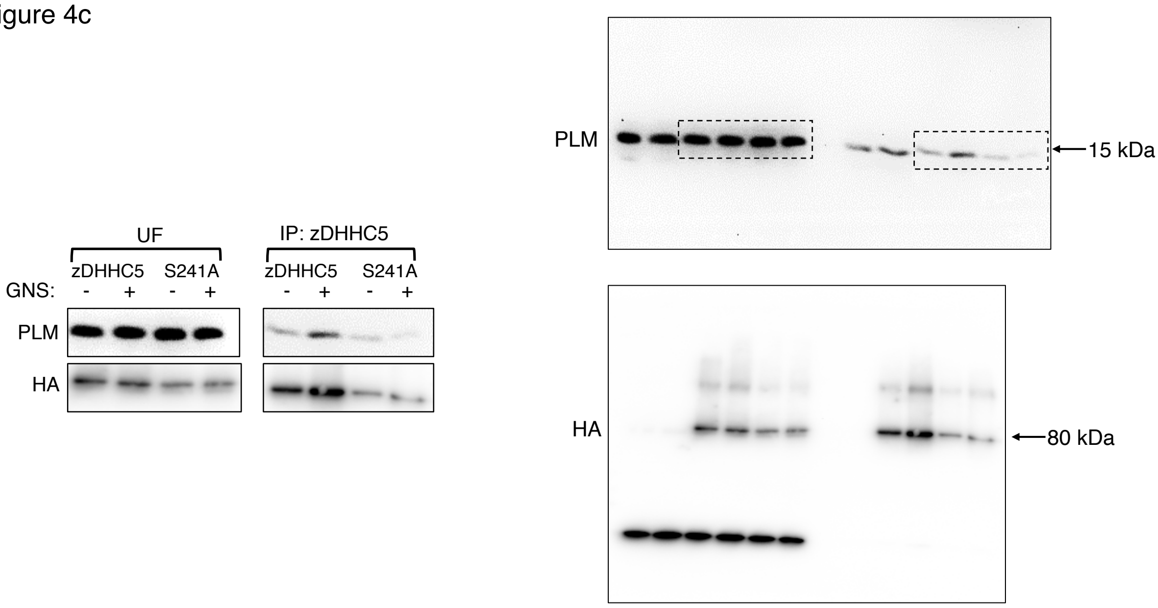

Figure 5a

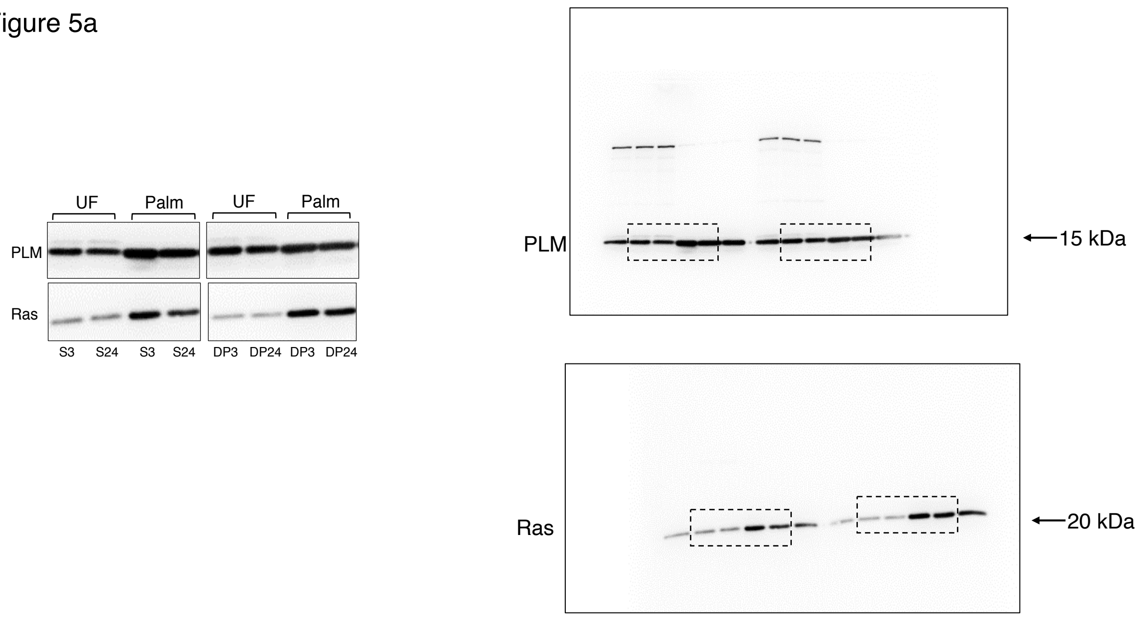

Figure 5b

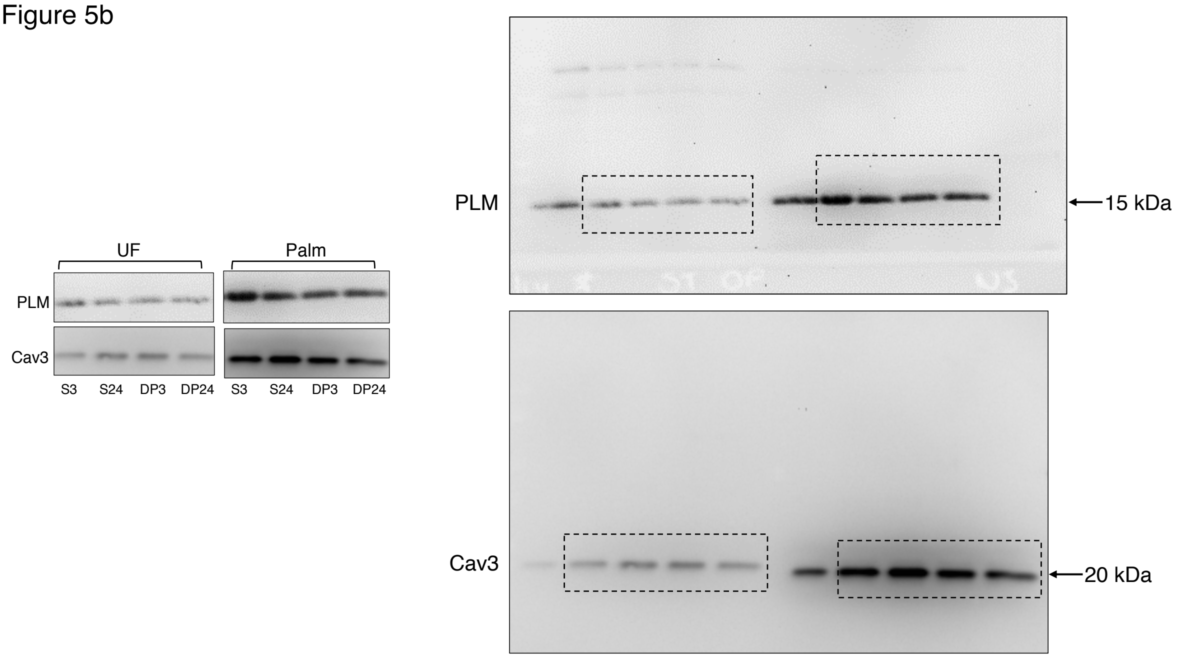

Supplement: Supplementary file 1 — Supplementary Information [file 42003_2020_1145_MOESM1_ESM.pdf]
