# Supplementary material for: Control of protein palmitoylation by regulating substrate recruitment to a zDHHC-protein acyltransferase
Source: Commun Biol. 2020 Jul 31;3:411. doi: 10.1038/s42003-020-01145-3 (PMC7395175; doi:10.1038/s42003-020-01145-3)
Supplement: Supplementary file 2 — Descriptions of Additional Supplementary Files [file 42003_2020_1145_MOESM2_ESM.pdf]

Descriptions of Additional Supplementary Files

Supplementary Data 1: Determination of zDHHC5 interactors in HEK cells by proximity biotinylation – protein IDs. List of proteins identified in pulldown experiments from HEK cells transfected with BirA alone (empty vector (EV)-BirA or BirA-EV) or BirA fused to zDHHC5 (DHHC5-BirA or BirA-DHHC5).

Supplementary Data 2: Determination of zDHHC5 interactors in HEK cells by proximity biotinylation – data analysis. High confidence zDHHC5 interactors were defined as those identified only in samples prepared from cells transfected with zDHHC5-BirA, BirA-zDHHC5 or both. Low confidence zDHHC5 interactors were identified as those present in both zDHHC5-BirA and BirA-zDHHC5 samples, but also in one sample from empty-vector (EV) transfected cells. Using SwissPalm 3, proteins were divided into palmitoylated (potential zDHHC5 substrates) and non-palmitoylated (potential zDHHC5 regulators). Analysis of both populations with DAVID 6.8 indicated enrichment of transmembrane adhesion molecules in the palmitoylated group with enrichment of protein-protein interaction motifs (PH, SH3, C2, PDZ, GRIP, PTB domains) in the non-palmitoylated group.

Supplementary Data 3: Determination of zDHHC5 interactors in H9C2 cells by proximity biotinylation High confidence zDHHC5 interactors were defined as those identified only in samples prepared from cells transfected with zDHHC5-BirA, BirA-zDHHC5 or both. Low confidence zDHHC5 interactors were identified as those present in both zDHHC5-BirA and BirA-zDHHC5 samples, but also in one sample from empty-vector (EV) transfected cells.

Supplementary Data 4: Source data underlying the graphs and charts presented in the main figures.
