# Supplementary material for: Control of protein palmitoylation by regulating substrate recruitment to a zDHHC-protein acyltransferase
Source: Commun Biol. 2020 Jul 31;3:411. doi: 10.1038/s42003-020-01145-3 (PMC7395175; doi:10.1038/s42003-020-01145-3)
Supplement: Supplementary file 7 — Reporting Summary [file 42003_2020_1145_MOESM7_ESM.pdf]

nature research

Corresponding author(s): William Fuller, Niall Fraser

Last updated by author(s): Jun 18, 2020

Reporting Summary

Nature Research wishes to improve the reproducibility of the work that we publish. This form provides structure for consistency and transparency in reporting. For further information on Nature Research policies, see Authors & Referees and the Editorial Policy Checklist.

Statistics

For all statistical analyses, confirm that the following items are present in the figure legend, table legend, main text, or Methods section.

n/a	Confirmed	
☐	☒	The exact sample size (*n*) for each experimental group/condition, given as a discrete number and unit of measurement
☐	☒	A statement on whether measurements were taken from distinct samples or whether the same sample was measured repeatedly
☐	☒	The statistical test(s) used AND whether they are one- or two-sided<br>*Only common tests should be described solely by name; describe more complex techniques in the Methods section.*
☐	☒	A description of all covariates tested
☐	☒	A description of any assumptions or corrections, such as tests of normality and adjustment for multiple comparisons
☒	☐	A full description of the statistical parameters including central tendency (e.g. means) or other basic estimates (e.g. regression coefficient) AND variation (e.g. standard deviation) or associated estimates of uncertainty (e.g. confidence intervals)
☒	☐	For null hypothesis testing, the test statistic (e.g. *F*, *t*, *r*) with confidence intervals, effect sizes, degrees of freedom and *P* value noted<br>*Give P values as exact values whenever suitable.*
☒	☐	For Bayesian analysis, information on the choice of priors and Markov chain Monte Carlo settings
☒	☐	For hierarchical and complex designs, identification of the appropriate level for tests and full reporting of outcomes
☒	☐	Estimates of effect sizes (e.g. Cohen's *d*, Pearson's *r*), indicating how they were calculated

Our web collection on statistics for biologists contains articles on many of the points above.

Software and code

Policy information about availability of computer code

Data collection	Western Blot data was acquired using QuantityOne 4.6.8 and Image Lab 4.0 (both BioRad)
Data analysis	Western Blot data was analysed using QuantityOne 4.6.8 and Image Lab 4.0 (both BioRad)

For manuscripts utilizing custom algorithms or software that are central to the research but not yet described in published literature, software must be made available to editors/reviewers. We strongly encourage code deposition in a community repository (e.g. GitHub). See the Nature Research guidelines for submitting code & software for further information.

Data

Policy information about availability of data

All manuscripts must include a data availability statement. This statement should provide the following information, where applicable:

- Accession codes, unique identifiers, or web links for publicly available datasets
- A list of figures that have associated raw data
- A description of any restrictions on data availability

Mass spectrometry data from proximity biotinylation experiments (MASCOT scores with all peptide IDs) are available in Supplementary Data 1-3

Field-specific reporting

Please select the one below that is the best fit for your research. If you are not sure, read the appropriate sections before making your selection.

☒ Life sciences ☐ Behavioural & social sciences ☐ Ecological, evolutionary & environmental sciences

For a reference copy of the document with all sections, see nature.com/documents/nr-reporting-summary-flat.pdf

Life sciences study design

All studies must disclose on these points even when the disclosure is negative.

Sample size	To observe a 25% difference between groups with a coefficient of variation of 0.1, power analysis indicates that the required sample size is 6 observations per group (using a 2-tailed t-test). Where larger differences occur between groups, a smaller sample size can be used. Here, sample sizes ranged between 3 and 6.
Data exclusions	No data was excluded from the analysis
Replication	All data presented has utilised numerous biological replicates. All data has been reproduced multiple times.
Randomization	Cultured cells were randomised to treatment groups in multi-well dishes at the time of plating
Blinding	Investigators were not blinded during data collection or analysis.

Reporting for specific materials, systems and methods

We require information from authors about some types of materials, experimental systems and methods used in many studies. Here, indicate whether each material, system or method listed is relevant to your study. If you are not sure if a list item applies to your research, read the appropriate section before selecting a response.

Materials & experimental systems

n/a	Involved in the study	
☐	☒	Antibodies
☐	☒	Eukaryotic cell lines
☒	☐	Palaeontology
☐	☒	Animals and other organisms
☒	☐	Human research participants
☒	☐	Clinical data

Methods

n/a	Involved in the study	
☒	☐	ChIP-seq
☒	☐	Flow cytometry
☒	☐	MRI-based neuroimaging

Antibodies

Antibodies used	Anti-PLM, Abcam Ab 76597, Lot# gr260084-1. Anti-sodium pump alpha1 subunit, Development Studies Hybridoma Bank, clone a6F. Anti-zDHHC5, Sigma Hpa014670, Lot# A105284. Anti- HA tag, Roche / Sigma, clone 3F10. Anti-H-ras, Merck / Millipore 05.516, Lot# 2931107. Cav3 – BD Biosciences, Clone 26 . PLM phospho-S68, custom made (Wypijewski, K.J. et al. (2013) J. Biol. Chem. 288, 13808-13820)
Validation	All antibodies were validated in-house against over-expressed and (where available) knockouts of target proteins.

Eukaryotic cell lines

Policy information about cell lines

Cell line source(s)	A stable cell line expressing phospholemman was engineered in FT-293 cells (originally obtained from Invitrogen, described in Howie et al PNAS 111 (49) 17534-17539). The creation of a zDHHC5 KO cell line is described in the manuscript text.
Authentication	None of the cell lines used were authenticated.
Mycoplasma contamination	Cell lines were not tested for mycoplasma contamination.
Commonly misidentified lines (See ICLAC register)	None

Animals and other organisms

Policy information about studies involving animals; ARRIVE guidelines recommended for reporting animal research

Laboratory animals	Wistar rats
Wild animals	The study did not involve wild animals
Field-collected samples	The study did not involve samples collected from the field.

Ethics oversight	ll protocols involving animals were approved by the Animal Welfare and Ethics Review Boards at the Universities of Dundee and Glasgow. Rat cardiac tissues were collected post-mortem after sacrificing animals using a method designated Schedule 1 by the Animals (Scientific Procedures) Act 1986.

Note that full information on the approval of the study protocol must also be provided in the manuscript.
